# Sports nutrition knowledge, source of nutrition information and dietary consumption pattern of Ugandan endurance athletes: a cross-sectional study of the Sebei sub-region

**DOI:** 10.1186/s13102-025-01157-8

**Published:** 2025-05-02

**Authors:** Joshua Musau, Oluyemisi F. Folasire, Nonhlanhla S. Mkumbuzi

**Affiliations:** 1 Sports Management and Policy Development Program, The Pan African University Life and Earth Sciences Institute (including Health and Agriculture– PAULESI), 200132 Ibadan, Nigeria; 2 Department of Human Kinetics, Faculty of Education, University of Ibadan, 200284 Ibadan, Nigeria; 3 Department of Human Nutrition and Dietetics, College of Medicine, University of Ibadan, 200285 Ibadan, Nigeria; 4 Department of Family Medicine, University College Hospital (UCH), 200285 Ibadan, Nigeria; 5 Department of Sports, Exercise and Rehabilitation, Northumbria University, Newcastle upon Tyne,, UK; 6 Department of Human Movement Science, Nelson Mandela University, Gqeberha, , South Africa; 7 Department of Rehabilitation, Midlands State University, Gweru, Zimbabwe; 8 NtombiSports (PTY) Ltd, Cape Town, South Africa

**Keywords:** Athlete, Nutrition, Sports, Knowledge, Endurance, African athletes

## Abstract

**Background:**

Athletes’ nutrition knowledge depends on the sources of nutrition information they are exposed to and their social status. Currently, there is a dearth of literature on the nutrition knowledge of Ugandan athletes. This study assessed endurance athletes’ sports nutrition knowledge, sources of nutrition information, and their dietary consumption patterns.

**Methods:**

A cross-sectional descriptive study including 100 purposively selected (middle and long distance) athletes from four Athletic Management camps was conducted in Kapchorwa district, Uganda. Using an interviewer-administered questionnaire, trained research assistants collected athletes’ demographic information, knowledge, practices, sources of nutrition information and consumption patterns. ANOVA, Independent t-test, Chi-square analysis, and Binary logistic analyses were done using SPSS^®^ software version 23.0.

**Results:**

Most athletes *n* = 74 (74.0%) in this study were aged between 15 and 17 years with at least 2 years’ experience in athletics. Majority *n* = 68 (68.0%) of the athletes had good sports nutrition knowledge while *n* = 32 (32.0%) showed poor knowledge. About *n* = 88 (88.0%) knew that vitamin consumption can enhance recovery after training. Athletic trainers *n* = 49 (49.0%) and family/parents *n* = 28 (28.0%) were primary sources of nutrition information, while nutritionists/dietitians *n* = 5 (5.0%) and the internet *n* = 4 (4.0%) were less consulted. Cereals were the most consumed food group *n* = 72 (72.0%), followed by legumes and nuts *n* = 67 (67.0%) while the least consumed foods were milk/egg *n* = 33 (33.0%)), fish *n* = 25 (25.0%) and meat/poultry *n* = 13 (13.0%). Good sports nutrition knowledge was associated with adequate dietary practices (*p* = 0.009). Sports nutrition knowledge differed significantly with age group (F = 4.566, *p* = 0.013), and between female and male (F = 21.884, *p* < 0.000) athletes. Age group was a significant predictor (β = 1.925, Ex(β) = 6.853, C. I = 0.882–36.096, *p* = 0.023) of sports nutrition knowledge.

**Conclusions:**

In this study endurance athletes had good sports nutrition knowledge, and relied primarily on their coaches/trainers for sports nutrition information. It is recommended that athletic trainers be offered supplementary training in sports nutrition to ensure proper nutrition knowledge dissemination among athletes.

**Supplementary Information:**

The online version contains supplementary material available at 10.1186/s13102-025-01157-8.

## Introduction

Nutritional intake for athletes is an important part of their adequate nourishment and well-being [1]. Nutrition is a key component in improving an athlete’s adaptation to endurance training and its effects [2]. Endurance activities require extreme physical and mental strength from an athlete and such events include those lasting approximately 26 min to about 4 h [2]. These events are often associated with a high turnover of nutrients, consequently predisposing endurance athletes to a risk of nutrient depletion on the field of play [3]. Sports nutrition guidelines recommend macronutrient intake of about 45-65% carbohydrates, 20-35% fat, and 10-30% protein in their meals supplemented with micronutrients like vitamins C and D and Iron in deficiency cases [1, 2, 4], but Proper use of nutrition strategies and consumption of the right food quantities for the appropriate nutrients requires that athletes be knowledgeable on sports nutrition.

Sports nutrition knowledge (SNK) is an individual’s understanding of concepts that relate to how to maintain a healthy body mass; manage body fluid balance; and fuelling techniques before, during, and after training or competition; supplement use; and knowledge of nutrition for athletic performance [5]. Nutrition education interventions on athlete populations report positive impacts on athletes’ sports nutrition knowledge and attitudes toward nutrition [6, 7]. However, SNK can also be influenced by ones’ social status: income level, education level, and SNK can also vary with age and sex of an individual [8–12]. Sex as a biological factor dictates the food intake, energy, nutrient requirements, and metabolism resulting in uneven health outcomes between males and females [13–15]. Generally, females are always mindful of their body image as compared to males and are more likely to seek nutrition knowledge and counselling [16]. Reports from athletes and non-athlete populations give evidence of positive dietary changes associated with high nutrition knowledge scores [17, 18]. This is corroborated by a recent research on 24-hour ultramarathon athletes reported positive dietary changes among those who had high knowledge scores [19]. This implies that dietary practices based on limited knowledge and misconceptions can negatively impact health and, eventually athletic performance [11].

Data on elite African athletes’ sports nutrition knowledge especially in Uganda are generally scarce. However, some data are available for Kenyan elite endurance athletes, Ugandan recreational athletes, and Ugandan university athletes. These data shows that athletes have poor nutrition knowledge of supplementation and that their sources of nutrition information are often not credible [20–22]. Additionally, these athletes rarely consult with registered dietitians or sports nutritionists [22], which may further predispose them to misinformation and associated health risks [20].

Despite the elite level at which Ugandan endurance athletes compete and their successes at this level, sports nutrition knowledge, sources of nutrition information, and dietary consumption patterns (DCP) of Ugandan endurance athletes remain unclear. Therefore, this study sought to investigate sports nutrition knowledge, sources of nutrition information, and dietary consumption patterns of endurance athletes in the Sebei sub-region of Uganda.

## Methods and materials

### Study design

A cross-sectional, descriptive study of Ugandan endurance athletes in the Sebei sub-region of Uganda.

### Study site

This study was conducted in the Eastern region of Uganda which for years has produced the most dominant athletes on the global scene, especially in distance events with the 10,000 m world records claimed in the Tokyo Olympic Games 2020, and the Budapest World Championship 2023 [23].

This region hosts the Ugandan National High Altitude Training Centre (NHATC) situated 2,550 m above sea level and where the majority of Ugandan endurance athletes train [24].

### Population

The study population was approximately 10,000 athletes in the Sebei sub-region; namely, Kapchorwa, Kween, and Bukwo Districts.

### Sampling and sample size

A purposive sampling technique was used to select Kapchorwa among three [3] sporting districts and four [4] athletic management camps in Kapchorwa; and a pragmatic purposive approach to select 100 male and female athletes as study participants. The participants selected were middle-distance and long-distance athletes.

The minimum required sample for the study was determined by Yamane’s formulae [25] and calculated as below.

$$\:\text{n}\:=\:\text{N}/(1+\text{N}{\left(\text{x}\right)}^{2})$$

Whereby n = Sample size, N = Total population, x = Level of Precision, 1 = constant, *N* = 10,000 athletes, x = 10%.

*n* = 10,000/ (1+(10,000(1/100)).

*n* = 99.009, *n* = 99.09 which is approximately 100 respondents.

The calculated minimum required sample size was 100 athletes at a priori alpha level of 0.05.

### Inclusion and exclusion criteria

Ugandan Middle and long-distance athletes aged 15–35 who had been actively training within the camps were recruited for this study. Sick athletes, and those athletes who resided out of camps where they received extra personalized training were not included in our study.

### Data collection

Data were collected between October and November 2023 and this involved 100 endurance athletes: *n* = 31 (31.0%) females and *n* = 69 (69.0%) males. Master’s level trained research assistants used an adapted SNK questionnaire from Folasire et al., [26] to assess athletes’ sports nutrition knowledge in their training camps. The questionnaire had 48 questions in total: on demographics (8 items), sports nutrition knowledge (15 items), dietary practices (15 items), and sources of nutrition information (3 items). A semi-quantitative Food Frequency Questionnaire (7 items) assessed the dietary consumption patterns (DCP), and food charts of locally consumed foods in this region were displayed to help participants to recall food consumed from different groups. Responses were recorded as TRUE/FALSE for sports nutrition knowledge questions and nutrition practice questions while YES/NO for other sections. Only two questions 1 and 15 required a participant to note down their responses for the sports nutrition knowledge section. For the FFQ, responses were chosen in the order of consuming from a particular food group 1 and 2 times daily, 1–3 and 4–6 times a weekly.

On pilot testing, the questionnaire took approximately eight [8] minutes per athlete. The questionnaire in the current study obtained a Cronbach alpha of 0.74, comparable to that reported by the designer [26].

### Human ethics and consent to participate

All participants were pledged anonymity and confidentiality of the data collected. Informed consent was obtained from each participant above consenting age while for those under 18 years old, their parents and legal guardians gave informed consent for participation in our study. This was done after giving an explanation of our study objectives and answering all questions from the participants and guardians regarding the study.

### Data analysis and management

Quantitative data were entered into Microsoft Excel^®^ version 2016 (Microsoft Corporation) and were analysed using SPSS software version 23.0 (Chicago, SPSS Inc). Data were reported as percentages, means, frequencies, and statistics of inference such as One-way analysis of variance (ANOVA), Logistic regression, and Chi-square were performed on the data.

All correct responses of respondents were summed, and an average was computed to determine scores for the participants’ sports nutrition knowledge and practice. The overall score was determined by summing all correctly answered question items. The correct scores for all of the sports nutrition knowledge items were summed for individual responses and used to calculate the mean (M = 24.83 ± 1.881). A percentage was generated from the scores, classification of respondents done was differentiating those that scored below (poor knowledge) and above the average score (good knowledge).

For Dietary consumption patterns (DCP) of the respondents, responses from the FFQ were classified as Frequent; for food groups consumed 1 and 2 times daily and infrequent; for those food groups consumed 1–3 and 4–6 times in a week.

Chi-square analyses were performed to ascertain possible relationships between variables of interest, e.g., nutrition knowledge categories with dietary consumption patterns (DCP), and also to establish relationships existing between nutrition knowledge categories and dietary practices and demographic information of the athletes.

One way-analysis of variance (ANOVA) was used to test the variation of the sports nutrition knowledge among different age groups and competition levels of athletes. An independent student t-test was tested to identify possible differences in knowledge scores for the sex and income categories of the respondents. Logistic regression analysis was performed to identify which independent variable was the best predictor for nutrition knowledge among the athlete population. Significant socio-economic variables were coded as dummy variables computed and ran in the model and the most significant with close to *p* = 0.00 was considered the best predictor of knowledge. The significance level was set at *p* < 0.05.

## Results

### Social-demographic information

Of the 100 participants, *n* = 31 (31.0%) were female and *n* = 69 (69.0%) were male. The largest proportion *n* = 75 (75.0%) of athletes were aged between 15 and 17 years while the least number *n* = 12 (12.0%) was from the age category 25–35 years.

The highest level of education attained by *n* = 54 (54.0%) was secondary school, followed by *n* = 43 (43.0%) who attained primary school level of education and only *n* = 3 (3.0%) of athletes reported in this study had attained tertiary-level education. All *n* = 100 (100.0%) of respondents lived in rural areas (villages).

There were twice as many middle-distance *n* = 67 (67.0%) compared to long-distance *n* = 33 (33.0%) athletes. The majority *n* = 79 (79.0%) of the respondents reported having a monthly family income of less than Uganda Shillings (UGX) 130,000 while only *n* = 21 (21.0%) earned more than Uganda Shillings (UGX) 130,000 monthly as seen in Supplementary Table 1.

### Sports nutrition knowledge scores

Most *n* = 68 (68.0%) of the athletes scored above the average score (M = 24.83 ± 1.881), and had good knowledge scores on sports nutrition while *n* = 32 (32.0%) of the respondents had scores less than the average and hence classified as having poor knowledge of sports nutrition. Similarly, *n* = 44 (44.0%) of athletes had inadequate dietary practices with their nutrition practice score below the average score (M = 20.34 ± 1.130), while *n* = 56 (56.0%) had scores above the mean with adequate dietary practices.

### Sports nutrition knowledge

**Table 1:** Sports nutrition knowledge (SNK) among Ugandan endurance athletes from the Sebei sub-region (*N* = 100)

Questions	Correct *n* (%)	Incorrect *n* (%)
1) How many times should we eat in a day?	97 (97.0)	3(3.0)
2) Do you think foods rich in carbohydrates are the main sources of energy in the body?	100 (100.0)	0 (0.0)
3) As an athlete, my food intake should increase	95 (95.0)	5 (5.0)
4) Can the lack of iron in the diet result in fatigue, injury, and illness?	70 (70.0)	30 (30.0)
5) Do you think sports drinks are the best to replace body fluids lost on the field of play?	62 (62.0)	38 (38.0)
6) Are vitamins good sources of energy?	86 (86.0)	14 (14.0)
7) Alcohol consumption can negatively affect the absorption and utilization of nutrients?	24 (24.0)	76 (76.0)
8) Eating of snacks is as good as eating home-prepared foods?	32 (32.0)	68 (68.0)
9) Do you think foods rich in sugar, jam and honey are suitable sources of energy for athletes?	93 (93.0)	7 (7.0)
10) The last meal before a competition should be consumed at least 3 h before a competition	57 (57.0)	43 (43.0)
11) Males and females of the same group use up the same amount of energy during exercise	43 (43.0)	57 (57.0)
12) Fruits and vegetables are important sources of vitamins and minerals?	89 (89.0)	11 (11.0)
13) Vitamins can enhance recovery after competition	88 (88.0)	12 (12.0)
14) Do you think milk and milk products are good sources of calcium?	82 (82.0)	18 (18.0)
15) How many classes of nutrients do we have?	42 (42.0)	58 (58.0)

Most respondents *n* = 97 (97.0%) knew that we should eat at least three [3] times in a day while *n* = 3(3.0%) did not answer correctly. Only, *n* = 42(42.0%) our athletes knew the six classes of nutrients while the rest did not know. With regards to sources of energy, all *n* = 100 (100.0%) of the athletes knew that foods rich in carbohydrates are the main source of energy for the body. Nearly all *n* = 95 (95.0%) of the respondents had the right knowledge that as an athlete, food consumption should be higher than compared to the general population. The knowledge that iron deficiency in the diet can cause fatigue, illness, and injury was known by at least *n* = 70 (70.0%) while *n* = 30 (30.0%) were not knowledgeable. About *n* = 62 (62.0%) of athletes knew that sports drinks more likely aid rehydration when body fluids are lost in the field of competition; *n* = 86 (86.0%) knew that vitamins are not a good source of energy; *n* = 76 (76.0%) had good knowledge that drinking alcohol affects digestion. Slightly more than half *n* = 57 (57.0%) of the athletes knew that a pre-event meal should be consumed at least 3 h before the event. The majority of the athletes *n* = 88 (88.0%) responded correctly to the statement that vitamins can enhance recovery after competition. A detailed summary is in Table 1 above.

### Dietary practices

The study also found that *n* = 64 (64.0%) of the athletes had adequate Dietary Practices (DP) with score above the average DP score (M = 20.34 ± 1.13), while *n* = 36 (36.0%) had scores less than the average score and these participants had inadequate Dietary Practices (DP).

Table 2 below shows that *n* = 27(27.0%) of the endurance athletes in this study use supplements such as multivitamins, while *n* = 73 (73.0%) do not. Most respondents *n* = 97 (97.0%) consume fruits and vegetables often while *n* = 3 (3.0%) do not; *n* = 36 (36.0%) skipped the pre-event meal while *n* = 64 (64.0%) of them did not skip the pre-event meal.

**Table 2:** Dietary practices (DP) among Ugandan endurance athletes from the Sebei sub-region (*N* = 100)

Questions	Yes % (*n*)	No %(*n*)
1) Do you use supplements like multivitamin as an athlete?	27 (27.0)	73 (73.0)
2) I consume lots of fruits and vegetables	97 (97.0)	3 (3.0)
3) I skip meals before a competition or an event	36 (36.0)	64 (64.0)
4) I eat just before an event	63 (63.0)	37 (37.0)
5) I eat just after an event	84 (84.0)	16 (16.0)
6) I consume sports drinks every day during practice or when I feel dehydrated	49 (49.0)	51 (51.0)
7) I eat an adequate diet daily	88 (88.0)	12 (12.0)
8) I change my pattern of eating at the time of a competition	90 (90.0)	10 (10.0)
9) I always take my breakfast daily	97 (97.0)	3 (3.0)
10) I consume lots of water during and after training/competition	100(100.0)	0 (0.0)
11) I always eat at least one hour before training/competition	46 (46.0)	54 (54.0)
12) I prefer snacks to a special diet before training and competition	42 (42.0)	58 (58.0)
13) I eat at least three times daily	100(100.0)	0 (0.0)
14) I consume milk and milk products daily	48 (48.0)	52 (52.0)
15) I consume alcohol to enhance my performance	0 (0.0)	100(100.0)

### Dietary consumption pattern

**Fig. 1:**
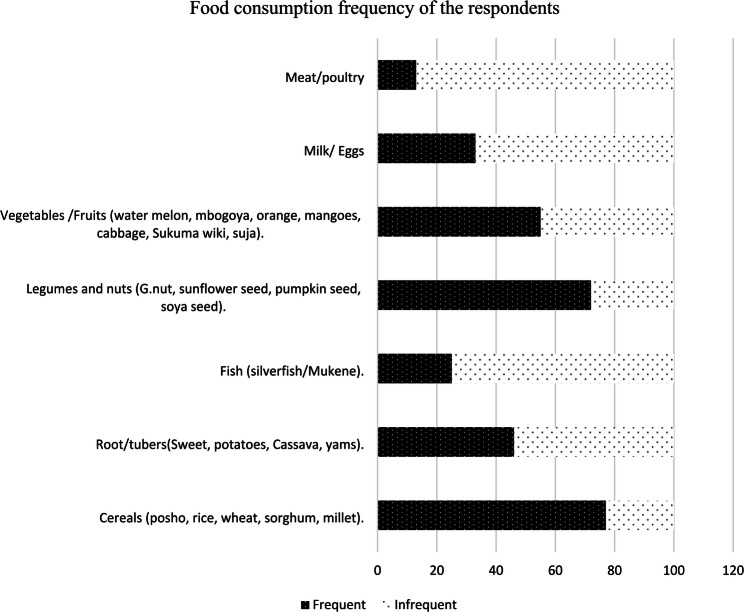
Dietary consumption frequency of Ugandan endurance athletes from the Sebei sub-region (*N* = 100) Dietary consumption pattern

Most athletes *n* = 77 (77.0%) frequently consumed from the cereal food group (e.g., posho, rice, wheat, sorghum, millet), *n* = 72 (72.0%) consumed from legumes/nuts group (e.g., groundnuts, sunflower seed, pumpkin seed, bean/soya seed), *n* = 55 (55.0%) consumed from vegetables/fruits group, (e.g., watermelon, Mbogoya, orange, mangoes, cabbage, Sukuma wiki, Suja), while *n* = 46 (46.0%) consumed from root/tubers group. The least consumed food groups were milk/egg *n* = 33 (33.0%), fish/Mukene *n* = 25 (25.0%) and meat/poultry *n* = 13 (13.0%) as seen in Fig. 1 below.

**Fig. 2:**
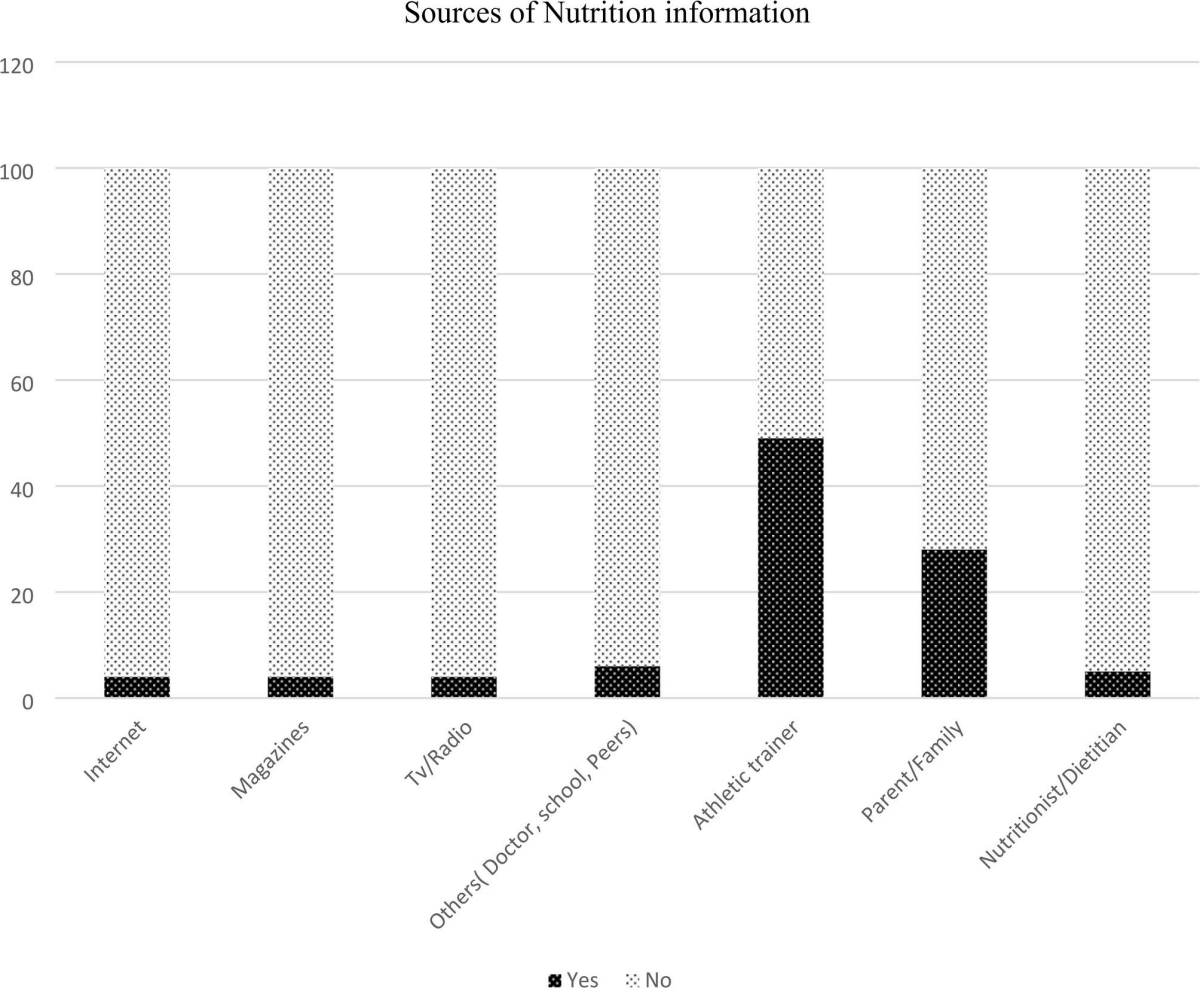
Sources of nutrition information among Ugandan endurance athletes from the Sebei sub-region (*N* = 100) Sources of nutrition information

As shown in Fig. 2 below, athletic trainers *n* = 49 (49.0%), parents/family *n* = 28 (28.0%), others (doctors, peers, school) *n* = 6 (6.0%), nutritionists/dietitians *n* = 5 (5.0%), magazines *n* = 4 (4.0%), TV/Radio *n* = 4 (4.0%) and the internet *n* = 4 (4.0%) were consulted for nutrition information. Only *n* = 9 (9.0%) of participants had previously undertaken a nutrition course.

### Sports nutrition knowledge and other variables

**Table 3:** Relationship between socio-demographic variables and SNK of Ugandan endurance athletes (*N* = 100)

Variable		Sports Nutrition Knowledge (SNK)		Chi-square $\:({x}^{2})$	*P* value
		Good	Poor		
		n (%)	n (%)		
Self-reported sex	Male Female	25(25.0) 7(7.0)	44(44.0) 24(24.0)	1.832	0.176
Age at recruitment	15–17 18–25 26–35	31(31.0) 1(1.0) 1(1.0)	43(43.0) 14(14.0) 11(11.0)	13.005	0.001**
Athletic event	Middle distance Long distance	26(26.0) 6(6.0)	41(41.0) 27(27.0)	4.322	0.038*
Duration of participation in athletics	1–2 3–5 > 5	19(19.0) 11(11.0) 2(2.0)	41(41.0) 16(16.0) 11(11.0)	2.600	0.272
Relationship status	Single Married Co-habiting Divorced	23(23.0) 5(5.0) 3(3.0) 1(1.0)	45(45.0) 15(15.0) 0(0.0) 8(8.0)	8.734	0.033*
The highest level of education attained	Primary Secondary Tertiary	14(14.0) 18(18.0) 0(0.0)	29(29.0) 36(36.0) 3(3.0)	1.462	0.481
Monthly family income (UGX)	0-130,000 > 131,000	31(31.0) 1(1.0)	48(48.0) 20(20.0)	9.063	0.003**

There was a significant relationship between SNK and athletes’ family monthly income (*p* = 0.003), relationship status (*p* = 0.033), age group (*p* = 0.001), and athletic event (*p* = 0.038), but not with the highest level of education (*p* = 0.481), sex of athlete (*p* = 0.176), and with number years of participation in athletics (*p* = 0.272). Table 3 above shows a summary of the results.

**Table 4:** Binary logistic analysis of significant socio-demographic variables of Ugandan athletes

Variable	Β	Ex(β)	*P*	C.I (95%)	
				Lower	Upper
Age group	1.925	6.853	0.023*	0.882	36.096
Athletic event	1.233	3.431	0.059	0.956	12.317
Relationship status	-0.880	0.415	0.047*	0.174	0.989
Monthly family income	2.010	7.461	0.065	0.882	63.144

Age was the best predictor for SNK (*p* = 0.023; C.I = 0.882–36.096). For every unit (1.925) increase in age, an athlete’s odds of being classified as knowledgeable were nearly seven (6.9) times. The model with all the significant socio-demographic variables accurately predicted those with good knowledge 85.0% of the time as seen in Table 4 above.

SNK was significantly associated with root tuber consumption (*p* < 0.05) but not with the rest of the food groups shown in Supplementary Tables 2 and there were no associations observed between SNK and sources of nutrition information. Also, having had a nutrition course previously did not correlate with nutrition knowledge (*p* = 0.159). All results are shown in Supplementary Table 3. Additionally, good SNK was significantly associated with adequate dietary practices (*p* = 0.009) as shown below in Table 5.

SNK was different between athletes of different sex (*p* < 0.000), with different respondents’ family income levels (*p* = 0.018), with different athletic events (*p* < 0.05), with different age groups (*p* = 0.013), and with different education levels of respondents (*p* = 0.024). SNK did not differ with athletes’ relationship status (*p* = 0.746) results in Table 6 below.

**Table 5:** SNK and DP score category and bivariate analysis for SNK and DP of Ugandan endurance athletes (*N* = 100)

Variable		Category			Respondents score				
					*n*			%	
SNK		Poor Good			32 68			32.0 68.0	
DP		Poor Good			44 56			44.0 56.0	
**Bivariate analysis**									
			SNK			$\:{x}^{2}$	df		*p*
DP			Poor	Good		6.895	1		0.009*
	Poor Good		8 24	36 32					

*Significant at *p* < 0.05

**Table 6:** Independent t-test analysis and ANOVA- analysis to determine variation in SNK within Ugandan athletes’ demographic (*N* = 100)

Variables			Mean	SD		F	t		*P*
Sex	Male Female		24.70 25.13	2.198 0.763		21.884	-1.067		0.000
Family monthly income	Low High		24.58 25.76	1.952 1.221		5.750	-2.630		0.018
Athletic event	Middle distance Long distance		24.81 24.88	2.210 0.927		21.601	− 0.181		0.000
**ANOVA-analysis**									
Variable		df			F			*P*	
Age group		2			4.566			0.013	
Relationship status		3			0.411			0.746	
Education Level		2			3.874			0.024	

## Discussion

From the findings, it is noted that male respondents constituted a bigger proportion than females. This is because generally, sports is strongly gendered in African settings and society tends to discourage females from participating in sports [27]. Our findings relate to a previous study by Baranauskas et al. [28], where male athletes were more than female athletes. The general trend of low participation of females in sports remains high, and this calls for strategies to accommodate more females in sports, for the case of our study incorporation of female sports staff (e.g. Coaches, Physiotherapists, and managers) could be a way to attract more females to sports. Additionally, we showed knowledge differences between the sexes, which differs from other studies such as Magee et al. [29],. Our observed sports nutrition knowledge differences with sex could be because female adolescents are generally aware of their body image and tend to seek nutritional counselling more than their male counterparts [16]. This also implies that there may be a need to provide sex-specific nutrition knowledge enhancement to athletes to accommodate this sex difference with more emphasis on male endurance athletes.

The majority of endurance athletes in this study were late adolescents and had participated for less than 2 years in athletics. This is similar to the cohort from Baranauskas et al., [28] whose respondents had a mean age of 17 years, but contrasts with a previous study conducted among Kenyan endurance athletes who were much older with the majority between 23 and 28 years of age with less than 3 years of participation, which is somewhat similar to our study findings [21]. The differences in age in our study, can be explained by the implementation of talent identification and development programs where athletes are sourced right from the primary school level and offered free welfare and training in camps. Unlike other countries like in Kenya where talent identification is still not fully implemented, we can observe more older athletes reported in their studies. Other studies reporting more older athletes could be a result of recruitment of professional athletes in their studies who have more years of experience.

The highest level of education attained by most athletes in our study was secondary school level, which is expected given their age and generally, such education levels are commonly reported in studies of Kenyan and Ugandan athletes [20, 21]. In Uganda, talent is seen as a source of livelihood and athletes who excel in athletics at early age tend to drop out of school not only because of underlying financial constraints but to focus more of their time on athletic training and competitions to earn income to support their families and siblings.

Our results showed differences in sports nutrition knowledge amongst athletes of different education levels. However, level of education has been associated with the SNK in other studies of physically active individuals [20, 21] and in our cohort may have implications for the development of future education materials, as these need to be tailored to the level of education of our cohort. Also, age and athletic category had a relationship with SNK which was not observed in a cohort of Kenyan endurance athletes [21]. Elsewhere, age had been reported to have a relationship with sports nutrition knowledge [30, 31]. This explains the fact that, younger athletes may have lesser experiences and maturity unlike their older counterpart who are more likely to have higher sports knowledge acquired over the years of competition and training. Athletic category of our cohort related with SNK, similarly to Burke et al., [2] they report that every athletic event comes with different nutritional needs. This implies that our athletes should receive tailored sports nutrition knowledge according to the athletic categories and not generic concepts.

The study found that most athletes had good dietary practices and were knowledgeable about when to eat and what to eat before and after training or competitions but a significant proportion of them didn’t know about the six classes of nutrients. This is similar to a study on undergraduate athletes from Nigeria where 63% of respondents reported adequate nutrition practices [26], as well as a study on Nepali athletes where 50% had good nutrition practice scores [31]. Female athletes displayed better dietary practices compared to males in the current study just as was observed in Koch et al.’s study of the Nemonit population [10]. Females generally are so prudent when it comes to what they eat, to keep tract of their body weight and sometimes adopt restrictive diets unlike their male counterparts. Since our endurance athlete’s nutrient requirements are usually greater than those of normal populations, females should be given special attention to avert the risk of Reduced energy deficiency (RED) and other common micronutrient deficiencies like Iron.

Almost 75% of the athletes did not skip meals while almost every athlete always ate their breakfast, as shown in other cohorts [32]. All respondents in this cohort take a lot of water during training and eat at least three meals a day. This is consistent with the findings of previous studies on in-training water consumption [26, 32] but contrasts with Oladunni & Sanusi [33], who reported that 77% of athletes from Nigeria skip meals. The present findings also concur with the findings of Folasire et al., [26], which reported that 82.7% of athletes ate a pre-event meal at least three hours before the event; 57% agreed that athletes’ food intake should be higher than non-athletes; 63.6% reported good knowledge of the six classes of nutrients.

Further, athletes in our study displayed a high level of sports nutrition knowledge when it came to the use of recovery strategies with 75% of endurance athletes having good knowledge of the consumption of vitamins to enhance recovery. This finding was corroborated by studies that show that vitamin intake enhances muscle recovery by negating the effects of exercise-induced muscle damage [34, 35]. Such recovery strategies could be adopted by endurance athletes following this position statement [2]. The present results are also corroborated by previous studies that report good sports nutrition knowledge in 56% of Kenyan endurance athletes and 69.42% of Ghanaian athletes [21, 36]. However, the current study contrasts the findings of Klein et al., [32] that reported low sports nutrition among their athletes. This current study further disagrees with a more recent study of young athletes that reported poor average sports nutrition knowledge of its’ male and female respondents [29]. Generally, the differences in the sports nutrition knowledge could be attributed to the different education levels, competition levels, and the access to nutrition education by participants in the different studies reviewed. From our study, good sports nutrition knowledge can be attributed to the role of the athletic trainers in guiding athletes in the camps, since the camps cannot afford the costs that come with employment of nutrition professionals.

Our study found a statistically significant relationship between athletes’ sports nutrition knowledge and dietary practice which correlates with a review that established that nutrition knowledge had a weak positive relationship with positive dietary changes [17]. Even though our results did not show a relationship between previously attending a nutrition course with sports nutrition knowledge, this phenomenon of ‘when we know better, we do better’ has also been demonstrated in other studies on sports nutrition knowledge [32, 37], and in other health domains, such as good menstrual hygiene and child caregiving where higher literacy is associated with better behaviours [38, 39]. Hence, it is important to enhance athletes’ sports nutrition knowledge. It is noteworthy, however, that in Uganda, Nassanga et al., [38] reported that a combination of factors interplay when it comes to how knowledge is translated into proper dietary practices. Primarily, poverty, food security, the attitudes of the individuals, and the nature of jobs people are engaged in are key determinants of nutritional knowledge transfer. In our cohort, in particular, the median household income was Uganda Shillings (UGX) 100,000 which was not only associated with Sports Nutrition Knowledge (SNK), but household income may not be enough to allow athletes purchase and consume all the food groups in the requisite amounts. Hence, despite Ugandan endurance athletes being knowledgeable about sports nutrition, they may not have the resources available to practice optimum sports nutrition due to low socioeconomic status [40]. Also, we assert that the family income of our athletes does not allow most athletes subscribe to specialised paid services in the form of nutritional counselling which leaves out the economically disadvantaged.

Concerning the source of nutrition information, the majority of the respondents identified athletic trainers as a popular source of knowledge used in the camps, followed by parents/ family. Nutritionists/Dietitians, internet, TV/Radio, magazines, and others like doctors, peers, and schools were rarely consulted for sports nutrition information. These study findings are similar to other studies, which found that coaches/trainers are popular sources athletes rely on for nutrition knowledge [20, 26, 36, 41, 42]. However, this contrasts with Kathure et al., [21] who showed that 60% of their Endurance athletes relied on the Internet as the main source of nutrition information. Further, findings from UK and Malaysian athletes show that 33–82% of them relied on internet/web sources for nutrition information [43, 44]. Interestingly, peer-reviewed literature was identified as the main source of information by Hispanic ultra-endurance athletes [45], while nutritionists and dietitians were popular sources amongst athletes from Australia [46].

In our cohort, less than a tenth consulted a nutritionist for nutrition information. This result was expected because of the lack of nutritionists/dietitians in athlete support staff in low and middle-income countries (LMICs) such as Uganda, due to inadequate budgets to cover the cost of such specialized staff [20]. Also, there exists a gap in the enrolment and training of nutritionists/dietitians within tertiary institutions in low and middle-income countries (LMICs) [47, 48]. In Uganda, the majority of graduate nutritionists and dietitians from tertiary institutions enter the job market with incompetencies in some crucial domains of practice [49]. In contrast, high-income countries (HICs) tend to have specialized and rigorously licensed and certified athlete nutrition staff [50, 51]. Access to credible knowledge from nutritionists/dietitians could help inform dietary changes that in turn promote the healthy nutritional status of athletes [52, 53] and reduce the likelihood of misinformation [4, 54]. Since African athletes rely primarily on their coaches/trainers for nutrition knowledge, it may be prudent to incorporate nutrition education in coach education [55, 56] as well as for athletes themselves [57]. Moreover, the earlier this nutrition education is done, the sooner it will shape eating habits that are carried on into their careers and adulthood [58]. From the literature, there exists an increasing use of unreliable sources of nutrition information by athlete populations especially: internet and coaches. With the popularity and convenience of the internet, unguided use of internet for nutrition information could potentially expose athletes to misinformation. Therefore, our athletes could benefit from web sources with recommendations by nutrition professionals. Athletic trainers could also benefit from supplementary training on the core practical concepts of sports nutrition to support their athletes in routine training and competitions.

In our cohort different food groups were consumed with varying frequencies, with high frequencies of consumption observed in the cereal and legume food groups, while lower frequencies were recorded amongst protein source foods and fruits. consumed. The high cereal consumption is likely a reflection of the geographic location of the region, which is typically mountainous with no viable fishing grounds (lakes, swamps, and rivers). Fish is only obtained from markets distant from the region. Cereals like maize and millet are readily available in this region from cultivation as are legumes and nuts. This variation in the frequency is related to the accessibility of food by athletes [59]. A previous report on dietary diversity supports findings of the current study where high frequency of cereals consumption was observed by athletes as cereals (maize, wheat, and millet) make up a big part of the diet of inhabitants in Eastern, Northern and as well in the Southern African regions [60].

Our cohort showed a low frequency of meat/poultry, fish/make, and milk/eggs consumption by endurance athletes. This low consumption of animal protein may indirectly translate into inadequate protein intake in this population. Similarly reported by Moss et al., protein needs were not met by their endurance athletes, especially female athletes [61]. Inadequate protein intake in endurance training compromises protein synthesis, compromises the immune system, promotes muscle atrophy, and prolongs recovery [62, 63]. In contrast, Lin et al. show evidence of enhanced endurance training with adequate protein intake [64]. Endurance athletes require a recommended daily allowance (RDA) of protein in a range not greater than 1.6 g protein per kg ( PRO/kg) of body weight to maintain lean muscle mass [65, 66]. It is imperative that our endurance athletes consume the RDA of proteins in their diets, for optimum nutritional status and general health and wellbeing.

In the current study, root/tubers (e.g., sweet, potatoes, cassava, yams) were not consumed as highly as reported in other studies [33]. Overall, the frequency of food group consumption is different from the profile observed among Nigerian athletes whose most frequently consumed food groups were, fish/meat, milk, and eggs (70.0– 80.0%), and the least frequently consumed food groups were legumes-nuts (25.0%) and healthy root-tubers (15.0%) [26]. Similar to a study on student athletes in Nigeria, knowledge positively related to the consumption of root/tubers like yams in Nigeria [33], SNK in our cohort was significantly and positively associated with root /tuber consumption. However, other studies have not observed the same [10]. Kamande et al., [67] found a strong positive association between knowledge and legume and fruit consumption of Kenyan athletes unlike in the present study. This implies that Ugandan endurance athletes have a tendency to consume tuber/roots that are a key component in endurance carbohydrate fuelling techniques, and special attention could be put on the timing strategies and frequency of consumption before and during competition.

### Recommendations

Since the athletes in this study often consult athletic trainers for nutrition knowledge, the National Council of Sports Uganda should develop routine capacity-building programs to offer supplementary Sports nutrition training to trainers. This knowledge in turn will hopefully be disseminated to endurance athletes in training sessions in their camps. In the long term, it is important that nutritionists/dietitians be engaged as part of athlete support personnel to ensure good SNK as well as optimal consumption of nutrients in Ugandan endurance athletes.

Management of athletic camps to incorporate protein-rich diets in athletes’ daily and weekly meal plans for optimal protein intake.

### Limitations and suggestions for future studies

This study is not without limitations. Firstly, the cohort only included 100 young endurance athletes from Sebei, with a few years of experience in athletics. Hence, the results should be generalized to other Ugandan regions and age cohorts with caution.

Secondly, the study did not objectively assess athletes’ dietary intake but relied on self-reported practices and food consumption frequency from different food groups. This has the inherent weaknesses of recall bias, social desirability bias, as well as inaccuracy. Studies that prospectively assess the adequacy of nutrient intake by this population are suggested.

## Conclusion

This study concluded that the majority of the athletes had good knowledge of sports nutrition and adequate dietary practices. Sex differences in SNK and dietary practices were observed in this cohort, which may have implications for the development of sex-specific sports nutrition educational materials. The intake of protein-rich food was inadequate, as shown by the low frequency of meat, fish, eggs, and poultry consumption. This may have implications for injury risk and athletic performance. The athletic trainer was identified as the most frequently consulted source of SNK in the camps and nutritionists/dietitians/doctors were rarely consulted. Therefore, Sports Nutrition should be included in the training of coaches/athletic trainers.

## Electronic supplementary material

Below is the link to the electronic supplementary material.

Supplementary Material 1

Supplementary Material 2

## Data Availability

Data presented in this study are availed upon request to the corresponding author at: musaujoshua2017@gmail.com.

## Abbreviations

SNK: Sports Nutrition Knowledge
DCP: Dietary Consumption Pattern
DP: Dietary Practice
