# Supplementary material for: Sports nutrition knowledge, source of nutrition information and dietary consumption pattern of Ugandan endurance athletes: a cross-sectional study of the Sebei sub-region
Source: BMC Sports Sci Med Rehabil. 2025 May 2;17:110. doi: 10.1186/s13102-025-01157-8 (PMC12046639; doi:10.1186/s13102-025-01157-8)
Supplement: Supplementary file 1 — Supplementary Material 1 [file 13102_2025_1157_MOESM1_ESM.docx]

**SECTION I: DEMOGRAPHIC INFORMATION.**

**Code:**

Instructions: complete the form below to the best of your knowledge

| QUESTION | RESPONSE |
| --- | --- |
| 1. What is your age? | ....................................... years |
| 1. What is your sex? | ☐ Man=1 ☐ Woman=2 |
| 1. Which athletic event do you practice? | ☐ Middle distance1 ☐ Long distance 2 |
| 1. How many years have you participated in athletics? | **Write here please.....................................** |
| 1. What is your relationship status? | ☐ Single (never married)=1  ☐ Married=2  ☐ Living together as married (co-habiting) =3  ☐ Divorced=4 |
| 1. What is the highest level of education/training you finished? | ☐ Primary school 1  ☐ Secondary school. (S. S) 2  ☐ Tertiary (University/Diploma/certificate)3 |
| 1. What is your family monthly income? | ☐ 0-130,000 UGX 0  ☐ >130,000UGX 1 |
| 1. Where do you live with your family? | ☐ Urban/ Town 1  ☐ Rural/Village 2 |

**SECTION II: SPORT NUTRITION KNOWLEDGE**

**Instructions:** This questionnaire consists of (14) fourteen closed items questions that assess your nutrition knowledge.

Please answer each question by placing an **(X)** in the appropriate column.

| QUESTION ITEMS | TRUE | FALSE |
| --- | --- | --- |
| 1. How many times should we eat in a day? **Write in gap** | 3 | |
| 1. Do you think foods rich in carbohydrates are the main sources of energy in the body? | 2 | 1 |
| 1. As an athlete, my food intake should increase | 2 | 1 |
| 1. Can lack of iron in the diet result in fatigue, injury and illness? | 2 | 1 |
| 1. Do you think sports drinks are the best to replace body fluids lost on the field of play? | 2 | 1 |
| 1. Are vitamins good sources of energy? | 1 | 2 |
| 1. Alcohol consumption can negatively affect the absorption and utilization of nutrients? | 2 | 1 |
| 1. Eating of snacks is as good as eating home prepared foods? | 1 | 2 |
| 1. Do you think foods rich in sugar, jam and honey are suitable sources of energy for athletes? | 2 | 1 |
| 1. The last meal before a competition should be consumed at least 3 hours before a competition | 2 | 1 |
| 1. Males and females of the same group use up the same amount of energy during exercise | 1 | 2 |
| 1. Fruits and vegetables are important sources of vitamins and minerals? | 2 | 1 |
| 1. Vitamins can enhance recovery after competition | 2 | 1 |
| 1. Do you think milk and milk products are good sources of calcium? | 2 | 1 |
| 1. How many classes of nutrients do we have? **Write in gap** | ........6...................... | |

**(Source: Adopted from Folasire et al, 2015)**

**SECTION III: DIETARY PRACTICE**

**Chose the correct response for the statements/questions below**

| QUESTION ITEMS | YES | NO |
| --- | --- | --- |
| 1. Do you use supplements like multivitamin as an athlete? | 1 | 0 |
| 1. I consume lots of fruits and vegetables | 1 | 0 |
| 1. I skip meals before a competition or an event | 0 | 1 |
| 1. I eat just before an event | 1 | 0 |
| 1. I eat just after an event | 1 | 0 |
| 1. I consume sports drinks every day during practice or when I feel dehydrated | 1 | 0 |
| 1. I eat adequate diet daily | 1 | 0 |
| 1. I change my pattern of eating at the time of a competition | 1 | 0 |
| 1. I always take my breakfast daily | 1 | 0 |
| 1. I consume lots of water during and after training/competition | 1 | 0 |
| 1. I always eat at least one hour before training/competition | 0 | 1 |
| 1. I prefer snacks to special diet before training and competition | 0 | 1 |
| 1. I eat at least 3 times daily | 1 | 0 |
| 1. I consume milk and milk products daily | 1 | 0 |
| 1. I consume alcohol to enhance my performance | 0 | 1 |

**(Source: Adopted from Folasire et al., 2015)**

**SECTION IV: SOURCE OF NUTRITION INFORMATION**

**Instructions: Chose the appropriate response by placing an (X) for the responses below**

| QUESTION | RESPONSE |
| --- | --- |
| 1. Do you have sports nutritionists/dieticians for nutrition services in your camp? | ☐ Yes 1  ☐ No 2 |
| 1. Have you had any previous nutrition courses? | ☐ Yes  ☐ No |
| 1. Which source of nutrition information do you use frequently? | - 1. Parents/Family   ☐ Yes 1  ☐ No 2   - 1. Magazine   ☐ Yes  ☐ No   - 1. TV/Radio   ☐ Yes  ☐ No   - 1. Athletic Trainer   ☐ Yes  ☐ No   - 1. Internet   ☐ Yes  ☐ No   - 1. Nutritionist/Dietitian   ☐ Yes  ☐ No   - 1. Other Specify..................... |

**(Source Adapted from (Muwonge et al., 2017))**

**SECTION V: FOOD FREQUENCY QUESTIONNAIRE (FFQ)**

**Indicate the number of times you eat from the different food groups**

| FOOD GROUPS | 1 TIME DAILY | 2 TIMES DAILY | 1-3 TIMES WEEKLY | 4-6 TIMES WEEKLY |
| --- | --- | --- | --- | --- |
| 1. Cereals (posho, rice, wheat, sorghum, millet). | 4 | 3 | 2 | 1 |
| 1. Root tubers   (Sweet, potatoes, Cassava, yams). |  |  |  |  |
| 1. Meat /Poultry (beef, chicken). |  |  |  |  |
| 1. Fish (silverfish/Mukene). |  |  |  |  |
| 1. Vegetables /Fruits (water melon, Mbogoya, orange, mangoes, cabbage, Sukuma wiki, suja). |  |  |  |  |
| 1. Legumes and nuts (G.nut, sunflower seed, pumpkin seed, soya seed). |  |  |  |  |
| 1. Milk/Eggs. |  |  |  |  |
