# Supplementary material for: Sports nutrition knowledge, source of nutrition information and dietary consumption pattern of Ugandan endurance athletes: a cross-sectional study of the Sebei sub-region
Source: BMC Sports Sci Med Rehabil. 2025 May 2;17:110. doi: 10.1186/s13102-025-01157-8 (PMC12046639; doi:10.1186/s13102-025-01157-8)
Supplement: Supplementary file 2 — Supplementary Material 2 [file 13102_2025_1157_MOESM2_ESM.docx]

**Supplementary file**

**Supplementary Table 1: Demographic characteristics of Ugandan endurance athletes from the Sebei sub-region. (N= 100).**

| Variable | Frequency  n (%) |
| --- | --- |
| Age at recruitment (years)  15 – 17  18 – 25  26 – 35 | 74 (74.0)  14 (14.0)  12 (12.0) |
| Self-reported sex  Male  Female | 69 (69.0)  31 (31.0) |
| Athletic event  Middle Distance  Long Distance | 67 (67.0)  33 (33.0) |
| Duration of athletics participation  <2 Years  3–5 Years  >5 Years | 60 (60.0)  27 (27.0)  13(13.0) |
| Highest level of education attained  Tertiary education  Secondary school  Primary school | 3 (3.0)  54 (54.0)  43 (43.0) |
| Monthly family income  0-130,000 UGX  >131,000 UGX | 79 (79.0)  21 (21.0) |
| Relationship status  Single  Married  Co-habiting  Divorced | 68 (68.0)  20 (20.0)  3 (3.0)  9 (9.0) |
| Area of residence  Urban  Rural | 0 (0.0)  100 (100.0) |

**Supplementary Table 2: Relationship between Sport Nutrition Knowledge (SNK) and Dietary Consumption Patterns (DCP) of Ugandan endurance athletes**.

| Food Group | Frequency | SNK | | Chi-square  $(x^{2}$) | P value |
| --- | --- | --- | --- | --- | --- |
|  |  | Good  n (%) | Poor  n (%) |  |  |
| Cereals (maize/posho, rice, wheat, millet) | Frequent  Infrequent | 51(51.0)  17(17.0) | 26(26.0)  6(6.0) | 0.480 | 0.488 |
| Root/tubers | Frequent  Infrequent | 45(45.0)  23(23.0) | 1(1.0)  31(31.0) | 34.826 | 0.000 |
| Meat/poultry | Frequent  Infrequent | 11(11.0)  57(57.0) | 2(2.0)  30(30.0) | 1.896 | 0.169 |
| Legumes and nuts | Frequent  Infrequent | 17(17.0)  51(51.0) | 8(8.0)  24(24.0) | 0.000 | 1.000 |
| Fish (Mukene) | Frequent  Infrequent | 40(40.0)  28(28.0) | 15(15.0)  17(17.0) | 1.252 | 0.263 |
| Vegetables/fruits | Frequent  Infrequent | 49(49.0)  19(19.0) | 23(23.0)  9(9.0) | 0.000 | 0.985 |
| Milk/eggs | Frequent  Infrequent | 22(22.0)  46(46.0) | 11(11.0)  21(21.0) | 0.040 | 0.841 |

**Supplementary Table 3: Relationship between SNK and sources of nutrition information of Ugandan endurance athletes (N=100).**

| Variable | Response | SNK | | Chi-square  $(x^{2}$) | P value |
| --- | --- | --- | --- | --- | --- |
|  |  | Good | Poor |  |  |
|  |  | n (%) | n (%) |  |  |
| Parent/family | Yes  No | 20(20.0)  48(48.0) | 8(8.0)  24(24.0) | 0.210 | 0.647 |
| Magazines | Yes  No | 1(1.0)  67(67.0) | 3(3.0)  29(29.0) | 3.541 | 0.06 |
| Tv/radio | Yes  No | 4(4.0)  64(64.0) | 0(0.0)  32(32.0) | 1.961 | 0.161 |
| Athletic trainer/coach | Yes  No | 34(34.0)  34(34.0) | 15(15.0)  17(17.0) | 0.085 | 0.771 |
| Internet | Yes  No | 3(3.0)  65(65.0) | 1(1.0)  31(31.0) | 0.094 | 0.75 |
| Nutritionist/dietitian | Yes  No | 2(2.0)  66(66.0) | 3(3.0)  29(29.0) | 1.896 | 0.168 |
| Others (school, doctor, peers) | Yes  No | 4(4.0)  64(64.0) | 2(2.0)  30(30.0) | 0.005 | 0.942 |
| Had nutrition course /Seminar | Yes  No | 8(8.0)  60(60.0) | 1(1.0)  31(31.0) | 1.983 | 0.159 |
